# Prevalence of Hyponatremia Among Adult Patients on Antidepressant Medications in a Tertiary Care Hospital: A Cross-Sectional Study

**DOI:** 10.7759/cureus.112321

**Published:** 2026-07-09

**Authors:** Pranav Goyal, Rajeshwari Gore, Shruti Sharma, Parika Kochhar

**Affiliations:** 1 Medicine, School of Medical Sciences and Research, Greater Noida, IND; 2 Pharmacology and Therapeutics, School of Medical Sciences and Research, Greater Noida, IND; 3 Psychiatry, Max Super Speciality Hospital, Noida, IND; 4 Psychiatry, Employees' State Insurance Corporation (ESIC) Medical College and Hospital, Noida, IND

**Keywords:** antidepressant medications, hyponatremia, prevalence, selective serotonin reuptake inhibitors, ssri

## Abstract

Introduction: This study aimed to estimate the prevalence of hyponatremia associated with antidepressant medications.

Methods: It was a cross-sectional observational study conducted for a duration of two months. Patients visiting the psychiatry department and fulfilling the inclusion criteria were recruited into the study after explaining the study and obtaining their written informed consent. Patient data was collected in a case report form and compiled on Microsoft Excel sheets (Microsoft Corp., Redmond, WA, USA). Patients displaying abnormal electrolyte levels were studied closely for association between the class of antidepressants prescribed to them, the individual drugs, the gender of the patient, and any concomitant medical illness or medications. Possible associations and correlations were documented.

Results: In our study sample, we observed that some patients taking the selective serotonin reuptake inhibitor (SSRI) class of antidepressant drugs displayed hyponatremia. Also, hyponatremia was seen only in the female patients of our study and not in the male patients. No association could be established with concomitant medical diseases or medications for developing hyponatremia in patients taking antidepressant drugs possibly due to the small sample size. Prevalence of hyponatremia was 2.65% with 95% CI in patients taking any antidepressant medication, while it was 3.5% in patients taking drugs of the SSRI class.

Conclusions: We conclude that more detailed and larger studies are required to establish an association between hyponatremia and antidepressant drugs, especially with SSRIs. Going forward, we suggest monitoring of serum sodium levels in patients who are prescribed antidepressant drugs.

## Introduction

Depression is a serious mental illness affecting a large number of people across the world. Studies have shown that nearly 282 million (5%) of adults and 64 million (5.7%) of the elderly population are affected by depression and approximately 3.8% (280 million people) of the world population is facing depression [1]. The COVID-19 pandemic has led to an upsurge and increased the caseload of depression. As per some studies, cases of anxiety and depression were reported by 291 million (41.3%) of adult patients suffering from COVID-19 [2].

Treatment of depression involves psychological counselling, use of antidepressant medications, and, in severe cases, use of electroconvulsive therapy. Among these, antidepressant medications are the most effective and the first choice of therapy in treating depression [3]. Commonly prescribed classes of antidepressant drugs are selective serotonin reuptake inhibitors (SSRIs), followed by selective norepinephrine reuptake inhibitors (SNRIs), along with some atypical antidepressants like bupropion and mirtazapine. There are also some older and now less commonly prescribed classes of drugs like tricyclic antidepressants (TCAs) and monoamine oxidase inhibitors (MAOIs) [4].

Reports of hyponatremia associated with the use of antidepressant medications have been surfacing since the last decade or so [5-7]. But comparatively fewer studies have been conducted to establish a correlation between antidepressant medication and hyponatremia.

Some theories do suggest that antidepressant intake can cause hyponatremia. According to one theory, antidepressant drugs inhibit the reuptake of serotonin which leads to a serotonin-induced increase in antidiuretic hormone (ADH), mediated by hypothalamic serotonin receptors [8]. Another theory suggests that both serotonin and noradrenaline can cause an increase in the ADH secretion by stimulating serotonergic and alpha-adrenergic receptors. An increase in ADH, in turn, increases thirst and water retention by binding to the kidneys' V2 receptors. Increased water reabsorption may be the reason to cause dilutional hyponatremia [9,10].

Antidepressant drugs cause many adverse reactions that are well documented and well known to us. Accordingly, the same are explained and conveyed to the patients at the time of consultation, and due precautions to avoid them are taken. To ensure adherence and positive therapeutic outcome, safe and effective antidepressant therapy is of utmost importance for patients suffering from depression. Not many studies have been done to clearly establish hyponatremia as an adverse reaction to antidepressant medications. Among available studies, very little data is available for the Indian population. We planned this study to understand or establish an association between antidepressant drugs and hyponatremia in the Indian population and contribute to help in preventing the complications associated with it.

## Materials and methods

It was an observational study, conducted cross-sectionally in the Department of Psychiatry in collaboration with the Department of Pharmacology of Sharda School of Medical Sciences and Research in Greater Noida, India. The duration of the study was two months. A total of 151 patients were recruited in the study, of whom 80 were female and 71 were male. The age of the patients included in the study ranged from 18 years to 79 years. Only three patients in the study group suffered from comorbidities like hypertension, diabetes, and hyperthyroidism. None of the patients had any renal diseases.

The inclusion criteria consisted of patients willing to participate in the study and give his/her informed consent and who were receiving antidepressant drugs prescribed by the Department of Psychiatry at our hospital for at least three weeks. We acknowledge that this could leave out the detection of early hyponatremia and possible discontinuation or hospitalization related to it. Patients not willing to give informed consent or who had suffered from vomiting or diarrhea within the last three days of recruitment were not included in the study.

The study was started after obtaining approval from the Institutional Ethics Committee of the School of Medical Sciences and Research and Sharda Hospital, Sharda University (approval number: SU/SMS&R/76-A/2O22/57). The convenience sampling method was used for our study. In total, 165 patients coming to the psychiatric outpatient and inpatient facilities were screened consecutively, and 158 patients were enrolled after they satisfied the inclusion criteria. Patients not willing to participate or not fulfilling the exclusion criteria were not included in the study. After obtaining written informed consent from the participants, relevant patient information was documented in a case report form (CRF). We encountered missing data in seven patients, and hence, only 151 patients were analyzed in our study. Serum electrolyte levels of all the enrolled patients were recorded. Patients showing serum sodium levels between 135 and 145 mEq/L were considered to have normal levels. Patients having serum sodium levels lower than 135 mEq/L were marked under the category of hyponatremia, and those having sodium levels more than 145 mEq/L were marked under the category of hypernatremia. Further, from the obtained patient data, the antidepressant drugs most commonly coinciding with hyponatremia were noted. Antidepressant drugs associated with hypernatremia were recorded. Any confounding factors contributing to hyponatremia were noted. All the data were then analyzed to estimate the hyponatremia among patients taking antidepressant medications. The identity of the patients remained confidential throughout the study, and all patient-related data were used only after obtaining informed consent from the patients. The number of patients exhibiting hyponatremia, hypernatremia, and normal sodium levels was documented.

For statistical analysis, the data was tabulated in the Microsoft Excel software (Microsoft Corp., Redmond, WA, USA) and analyzed through IBM SPSS Statistics for Windows, V. 25.0 (IBM Corp., Armonk, NY, USA). Sodium levels corresponding to various classes of antidepressants were documented. Then, through a comparative study, the most common drugs or groups of antidepressants causing hyponatremia were recorded. Any other factors like age, gender, or concomitant medications contributing to hyponatremia were noted. A sample size of 151 was calculated by using the Cochran formula $\mathrm{n}=(\mathrm{Z}^{2}\mathrm{Pq})/\mathrm{d}^{2}$.

Sample size

The sample size of the research population will be taken as 151 according to the Cochran formula $\mathrm{n}=(\mathrm{Z}^{2}\mathrm{Pq})/\mathrm{d}^{2}$. Here, Z is the confidence level which is taken as 95% (most reliable as it provides statistical reliability and practical feasibility). According to the Z table, Z=1.96. d is the desired level of precision (i.e., the margin of error). In this case, we have taken it to be 8% to achieve acceptable precision while remaining feasible within the available study population and resource constraints. p is a sample proportion or an estimated proportion of an attribute that is present in the population, which is taken as 0.5 (50%) due to the absence of prior prevalence data, the most conservative estimate. The value of q was calculated as 1-p; therefore, with p=0.50, q=0.50. n is the sample size. The overall prevalence of hyponatremia among patients treated with antidepressants was calculated, along with its 95% confidence interval (95% CI). Analysis was descriptive due to the low event count.

## Results

In our study, all the patients were receiving antidepressant medications for various conditions like depression, panic disorders, anxiety disorders, and dissociative disorders.

Table 1 presents the baseline characteristics of the study participants and the antidepressant medications used.

**Table 1:** Baseline demographic and clinical characteristics of the study participants and antidepressant medications used SSRIs: selective serotonin reuptake inhibitors; IPD: inpatient department; OPD: outpatient department; TCAs: tricyclic antidepressants; NaSSA: noradrenergic and specific serotonergic antidepressants; SNRIs: selective norepinephrine reuptake inhibitors

Variable	Category/value	N=total patients (%)
Age (years)	Mean±SD	38.84±14.47
Sex distribution	Male	71 (47%)
	Female	80 (53%)
Patient setting	IPD	100 (66%)
	OPD	51 (34%)
Antidepressant agent/class	SSRIs	114 (75%)
	TCAs	25 (16.5%)
	NaSSA	8 (5.2%)
	SNRIs	4 (2.6%)
SSRIs	Paroxetine	39 (34%)
	Escitalopram	37 (32%)
	Sertraline	27 (24%)
	Fluoxetine	11 (10%)
Comorbidities	Hypertension	2 (1.32%)
	Diabetes with hypertension	1 (0.66%)
	Renal disease	None
	Hyperthyroidism	None

Table 2 shows that the mean serum sodium levels ranged between 137.5 and 141.66 mEq/L. Amitriptyline had the highest and duloxetine had the lowest mean serum sodium levels. Sertraline, mirtazapine, and escitalopram have identical serum sodium levels (140.6 mEq/L), although their SD differed.

**Table 2:** Comparison of mean±SD of sodium levels of patients receiving antidepressant drugs

Patients receiving antidepressant drugs (%)	Mean±SD of sodium levels
Sertraline (18.5%)	140.6±3.20
Escitalopram (25%)	140.6±2.81
Paroxetine (25.4%))	140±6.09
Mirtazapine (7.3%)	140.62±1.18
Amitriptyline (5.3%)	141.66±2.27
Fluoxetine (15.9%)	140.36±2.20
Duloxetine (1.3%)	137.5±0.70
Venlafaxine (1.3%)	141±0

Table 3 shows the percentage of patients who developed hyponatremia while being treated with antidepressant drugs.

**Table 3:** Percentage of patients who developed hyponatremia during antidepressant therapy SSRIs: selective serotonin reuptake inhibitors

SSRIs	Patients developed hyponatremia (%)
Paroxetine	3/39 (7.6%)
Escitalopram	0/37
Sertraline	1/27 (3.7%)
Fluoxetine	0/11

Table 4 describes the serum electrolyte levels of the study subjects. In our study, the average serum sodium levels were 140.8 mEq/L. We observed that the majority of the patients had normal serum sodium levels (140 mEq/L), while four patients developed hyponatremia and seven patients developed hypernatremia. Similarly, the majority of the patients showed normal potassium levels, except for two patients who developed hypokalemia and three patients who developed hyperkalemia. Ninety-eight patients had normal serum chloride levels, while no patient developed hypochloremia and 53 patients developed hyperchloremia.

**Table 4:** Serum electrolyte levels of the patients in our study

	Number of patients
Normal serum sodium levels (135-140 mEq/L)	140
Hyponatremia	4
Hypernatremia	7
Normal serum potassium levels (3.5 and 5 mmol/L)	146
Hypokalemia	2
Hyperkalemia	3
Normal serum chloride levels (96-106 mEq/L)	98
Hypochloremia	0
Hyperchloremia	53

Table 5 shows the characteristics of patients who displayed hyponatremia during their treatment with SSRIs. Out of the four cases, three patients were taking paroxetine, and one patient was taking sertraline.

**Table 5:** Characteristics of the patients who developed hyponatremia SSRI: selective serotonin reuptake inhibitor

Patient no.	Age of the patient	Gender	Sodium levels of the patient (mEq/L)	Diagnosis of the patient	Drugs used	Class of the drug	Dose of the drug
1	46	Female	128	Anxiety	Sertraline	SSRI	25 mg
2	36	Female	133	Dissociative disorder	Paroxetine	SSRI	12.5 mg
3	24	Female	133	Depression	Paroxetine	SSRI	12.5 mg
4	61	Female	134	Depression	Paroxetine	SSRI	12.5 mg

Table 6 describes the cases of hypernatremia observed in our study. A total of seven patients developed hypernatremia, out of which five were taking drugs that belonged to the SSRI class of antidepressants and two were taking TCAs. Among the five patients taking SSRIs who developed hypernatremia, three were taking paroxetine and two were on escitalopram. The two patients who developed hypernatremia during treatment with TCAs were both on amitriptyline.

**Table 6:** Characteristics of the patients who developed hypernatremia SSRI: selective serotonin reuptake inhibitor; TCA: tricyclic antidepressant

Patient no.	Age of the patient	Gender	Sodium levels of the patient (mEq/L)	Diagnosis of the patient	Drugs used	Class of the drug	Dose of the drug
1	48	Female	147	Depression	Escitalopram	SSRI	10 mg
2	38	Female	146	Depression	Escitalopram	SSRI	10 mg
3	53	Female	147	Dysthymia and panic attack	Amitriptyline	TCA	25 mg
4	45	Male	147	Depression	Amitriptyline	TCA	25 mg
5	25	Male	151	Depression	Paroxetine	SSRI	12.5 mg
6	41	Male	147	Depression	Paroxetine	SSRI	12.5 mg
7	46	Male	152	Depression	Paroxetine	SSRI	12.5 mg

The prevalence of hyponatremia due to antidepressant medications in our study was 4/151 (2.65% with 95% CI) and specifically observed with SSRIs was 4/114 (3.5% with 95% CI).

## Discussion

Antidepressant therapy has been life-changing for numerous patients suffering from depression and associated disorders. It comes with its own set of complications, and preparing the patient to face them becomes an important part of the therapy for an overall positive outcome. A few studies have indicated an association between the use of antidepressant medications and the development of hyponatremia. However, awareness and seriousness about this are lacking, maybe due to the insufficient data and limited knowledge of the role of the confounding factors. Our study focused on finding the prevalence of hyponatremia among patients taking antidepressant medications. An attempt was made to improve our understanding of the factors contributing to the development of hyponatremia in our patients.

As per our study, we observed that hyponatremia was present in only those patients who were taking SSRIs and not in those who were taking any other class of antidepressant drugs. These findings were similar to a study conducted by Movig et al., who found that those taking SSRIs had a three times increased risk of developing hyponatremia [5]. As per their study, the other risk factors for hyponatremia were old age and abnormal potassium levels. They found that elderly patients (>65 years) had a six times increased risk of developing hyponatremia as compared to other age groups [5]. But in our study, old age was not considered a risk factor as out of the four cases of hyponatremia that we came across, three were below 60 years of age. This could also be due to the limited sample size and inclusion of all age groups in our study, as elderly patients comprised only 5.3% of our study population. Also, we did not find any association between abnormal potassium levels and hyponatremia, as all the cases that developed hyponatremia in our study had normal potassium levels (3.5-5.5 mEq/L). The percentage of hyponatremia with SSRIs as per the study by Letmaier et al. was 0.06% [11], whereas in our study, it was 2.65%.

Leth-Møller et al. [12], who studied the association of individual antidepressant drugs and hyponatremia in a Danish population, found that hyponatremia was associated with the use of most antidepressant drugs except for mianserin. They reported the strongest association between hyponatremia and citalopram, with the lowest association observed for duloxetine, venlafaxine, and mirtazapine. In our study, hyponatremia was observed among patients receiving paroxetine and sertraline; however, no drug-specific association could be established because of the limited sample size and the higher proportion of patients prescribed these medications compared with other SSRIs.

The electrolyte disturbances other than hyponatremia that were observed in our study were hypernatremia (4.6%) and hyperchloremia (35%). These findings were unique and could be purely incidental. Also, we could not find any studies where similar observations were reported in association with the use of antidepressant drugs.

Kirby et al. [13] in their study found that confounding factors like any medical illness and concomitant drugs did not pose an additional risk factor for developing hyponatremia in patients taking antidepressant medications. In our study, we also could not find a correlation between concomitant medications and medical illness with hyponatremia associated with antidepressant drugs as none of the cases that developed hyponatremia had any other medical disease or were taking any concomitant medications, but this could be due to the limited sample size, comorbidities, events, and lack of proper confounder measurement.

All the cases of hyponatremia in our study developed only in females; this observation was also seen in studies conducted by Giorlando et al. and De Picker et al., who stated that there was a higher incidence of hyponatremia associated with antidepressant use in females as compared to males [6,7].

The prevalence of antidepressant-induced hyponatremia as per our study was 2.65% for any antidepressant drug, whereas it was a little higher at 3.5% specifically for the SSRI class of antidepressant drugs. Meanwhile, in a study conducted by Mannesse et al., the prevalence of antidepressant-induced hyponatremia was higher (9.3%) than our study [14].

In our study, we attempted to evaluate the development of hyponatremia in patients treated with antidepressant medication. There are some limitations in our study including a small sample size and a lack of detailed coverage of all the contributing risk factors. More detailed studies with larger sample sizes are required to further improve our understanding of the causes and precautions to be undertaken to lower the risk of hyponatremia in patients taking antidepressant medications and make antidepressant therapy as safe as possible.

Limitations

We acknowledge the limitations of our study. As it was a part of a short-term studentship program, the sample size is small due to the short duration of the study period. We also recognize that the potential of selection bias and confounding factors could not be completely eliminated. Although demographic and clinical information was collected, not all factors known to influence serum sodium concentrations (such as concomitant medications, comorbid conditions, hydration status, or other causes of hyponatremia) could be comprehensively measured or controlled. Therefore, residual confounding may have influenced the observed association between antidepressant use and hyponatremia. In addition, we agree that our definition of hyponatremia was based solely on a serum sodium concentration of <135 mmol/L. This biochemical definition identifies the presence of hyponatremia but does not distinguish antidepressant-induced hyponatremia from hyponatremia due to other etiologies.

Accordingly, our findings should be interpreted as describing the occurrence of hyponatremia among patients receiving antidepressant therapy rather than proving that antidepressants were the direct cause of hyponatremia. Despite these limitations, the study provides valuable real-world data on the frequency of hyponatremia in antidepressant-treated patients and highlights the importance of routine serum sodium monitoring, particularly in individuals at increased risk. Future prospective studies incorporating comprehensive clinical evaluation and diagnostic work-up are warranted to better establish causality and identify the underlying mechanisms of antidepressant-associated hyponatremia.

## Conclusions

We found that hyponatremia was present in some patients treated with SSRIs. The prevalence of hyponatremia in our study was 2.65% with 95% CI for any class of antidepressant drug and 3.5% with 95% CI with SSRIs. Our study results are preliminary and should be approached with caution as per our acknowledged limitations. Due to the limited sample size, we could not establish any association with concomitant medications and medical conditions contributing to antidepressant-induced hyponatremia.

## References

[1] (2026). Depressive disorder (depression). https://www.who.int/news-room/fact-sheets/detail/depression.

[2] Pérez-Cano HJ, Moreno-Murguía MB, Morales-López O, Crow-Buchanan O, English JA, Lozano-Alcázar J, Somilleda-Ventura SA (2020). Anxiety, depression, and stress in response to the coronavirus disease-19 pandemic. Cir Cir.

[3] Kennedy SH (2013). A review of antidepressant therapy in primary care: current practices and future directions. Prim Care Companion CNS Disord.

[4] (2026). Commonly prescribed antidepressants and how they work. https://magazine.medlineplus.gov/article/commonly-prescribed-antidepressants-and-how-they-work.

[5] Movig KL, Leufkens HG, Lenderink AW, van den Akker VG, Hodiamont PP, Goldschmidt HM, Egberts AC (2002). Association between antidepressant drug use and hyponatraemia: a case-control study. Br J Clin Pharmacol.

[6] Giorlando F, Teister J, Dodd S, Udina M, Berk M (2013). Hyponatraemia: an audit of aged psychiatry patients taking SSRIs and SNRIs. Curr Drug Saf.

[7] De Picker L, Van Den Eede F, Dumont G, Moorkens G, Sabbe BG (2014). Antidepressants and the risk of hyponatremia: a class-by-class review of literature. Psychosomatics.

[8] Liu BA, Mittmann N, Knowles SR, Sheer NH (1996). Hyponatremia and the syndrome of inappropriate secretion of antidiuretic hormone associated with the use of selective serotonin reuptake inhibitors: a review of spontaneous reports. CMAJ.

[9] Brownfield MS, Greathouse J, Lorens SA, Armstrong J, Urban JH, Van de Kar LD (1988). Neuropharmacological characterization of serotoninergic stimulation of vasopressin secretion in conscious rats. Neuroendocrinology.

[10] Leibowitz SF, Eidelman D, Suh JS, Diaz S, Sladek CD (1990). Mapping study of noradrenergic stimulation of vasopressin release. Exp Neurol.

[11] Letmaier M, Painold A, Holl AK (2012). Hyponatraemia during psychopharmacological treatment: results of a drug surveillance programme. Int J Neuropsychopharmacol.

[12] Leth-Møller KB, Hansen AH, Torstensson M (2016). Antidepressants and the risk of hyponatremia: a Danish register-based population study. BMJ Open.

[13] Kirby D, Harrigan S, Ames D (2002). Hyponatraemia in elderly psychiatric patients treated with selective serotonin reuptake inhibitors and venlafaxine: a retrospective controlled study in an inpatient unit. Int J Geriatr Psychiatry.

[14] Mannesse CK, Jansen PA, Van Marum RJ, Sival RC, Kok RM, Haffmans PM, Egberts TC (2013). Characteristics, prevalence, risk factors, and underlying mechanism of hyponatremia in elderly patients treated with antidepressants: a cross-sectional study. Maturitas.

